# Household Pet Ownership and the Microbial Diversity of the Human Gut Microbiota

**DOI:** 10.3389/fcimb.2020.00073

**Published:** 2020-02-28

**Authors:** Ashley E. Kates, Omar Jarrett, Joseph H. Skarlupka, Ajay Sethi, Megan Duster, Lauren Watson, Garret Suen, Keith Poulsen, Nasia Safdar

**Affiliations:** ^1^Division of Infectious Disease, Department of Medicine, School of Medicine and Public Health, University of Wisconsin-Madison, Madison, WI, United States; ^2^Department of Medicine, William S. Middleton Veterans Hospital Madison, Madison, WI, United States; ^3^Department of Bacteriology, College of Agricultural and Life Sciences, University of Wisconsin-Madison, Madison, WI, United States; ^4^Department of Population Health Sciences, School of Medicine and Public Health, University of Wisconsin-Madison, Madison, WI, United States; ^5^Department of Medical Sciences, School of Veterinary Medicine, University of Wisconsin-Madison, Madison, WI, United States

**Keywords:** 16s rRNA sequencing, cats, dogs, cross-sectional design, epidemiology

## Abstract

The human gut microbiome has a great deal of interpersonal variation due to both endogenous and exogenous factors, like household pet exposure. To examine the relationship between having a pet in the home and the composition and diversity of the adult gut microbiome, we conducted a case-control study nested in a larger, statewide study, the Survey of the Health of Wisconsin. Stool samples were collected from 332 participants from unique households and analyzed using 16S rRNA sequencing on the Illumina MiSeq. One hundred and seventy-eight participants had some type of pet in the home with dogs and cats being the most prevalent. We observed no difference in alpha and beta diversity between those with and without pets, though seven OTUs were significantly more abundant in those without pets compared to those with pets, and four were significantly more abundant in those with pets. When stratifying by age, seven of these remained significant. These results suggest that pet ownership is associated with differences in the human gut microbiota. Further research is needed to better characterize the effect of pet ownership on the human gut microbiome.

## Introduction

The human gastrointestinal tract is home to a wide array of different microorganisms and their associated genes—known as the microbiome—which has a major influence on health and disease. The gut microbiome may be influenced by both endogenous and exogenous factors. In recent years, there has been increasing interest in what environmental factors influence and alter the composition of the microbiome over the human lifespan. One such environmental exposure of interest is pet ownership.

Research from the 1980s demonstrated that pets and their owners share common intestinal bacteria (Caugant et al., 1984). Studies in infants and young children have shown that early life exposure to household furry pets increases richness and diversity of the human gut microbiome (Azad et al., 2013; Tun et al., 2017). Moreover, exposure to pets is known to decrease the rate of atopic and allergic disease (Hesselmar et al., 1999; Litonjua et al., 2002; Ownby et al., 2002) and may also reduce the risk of metabolic diseases (Tun et al., 2017). Pet ownership has been hypothesized to act on the adult gut (Song et al., 2013) and skin (Damborg et al., 2009) microbiome through physical contact with pets and pet feces, which may be more frequent in adulthood.

To test the hypothesis that adults with at least one indoor pet have a different gut microbiota than those without a pet present in the home, we conducted a nested case-control study examining the effects of pet exposure on the adult gut microbiota.

## Materials and Methods

### Study Design and Population

We conducted a case-control study on the impact of indoor pet exposure on the human gut microbiota nested within the Survey of the Health of Wisconsin (SHOW) and its ancillary study, the Wisconsin Microbiome Study (WMS) (Eggers et al., 2018). Both SHOW and WMS are ongoing studies assessing the overall health and risk of infection with antibiotic-resistant organisms. The SHOW enrolls families from across the state of Wisconsin using census blocks.

All participants are over 18 years of age, capable of giving informed consent, able to communicate answers to the interviewers, and are not residents of nursing homes, hospitals, mental health institutions, penal institutions, or full-time members of the armed forces. The full list of inclusion and exclusion criteria as well as a description of the SHOW and the WMS populations has been published elsewhere (Nieto et al., 2010; Eggers et al., 2018). A description of the SHOW and WMS projects as well as SHOW protocols can be found on the SHOW website (https://www.show.wisc.edu). For this study, we included one participant from each household included in the SHOW and the WMS during 2016. To be included in the dataset, the participant had to have provided a stool sample. The SHOW survey asks if there are any of the following pets in the home: dogs, cats, birds, rodents, or reptiles, in addition to the number of resident pets. Additional information collected by the SHOW includes social and environmental factors, quality of life, economic factors, health care access, and health determinants. In addition to information collected for the SHOW, the WMS collects stool specimens and participants answer questions on infection history, medications, and diet. All study procedures were approved by the Institutional Review Board at the University of Wisconsin-Madison.

### Microbiota Analysis

A detailed description of sample collection, DNA extraction and amplification has been published elsewhere (Eggers et al., 2018). Briefly, DNA was extracted using a bead-beating protocol with an additional enzymatic lysis containing mutanolysin, lysostaphin, and lysozyme to help lyse Gram-positive cell walls. The V4 region of the 16S rRNA gene was sequenced on an Illumina MiSeq using 2 ×250 paired-end reads at the University of Wisconsin Biotechnology Center. Laboratory negative controls were used during each step of extraction and sequencing. A description and characterization of the negative controls can be found in the S1. Text.

Sequences were processed and binned into operational taxonomic units (OTUs) using the USEARCH/UPARSE pipeline (Edgar, 2013) as well as VSEARCH (Rognes et al., 2016). OTUs were classified to the genus level whenever possible using the RDP database (Cole et al., 2014). The Shannon (1948) and Inverse Simpson's (1949) diversity indices were used to assess alpha diversity. The Bray-Curtis dissimilarity matrix (Bray and Curtis, 1957) was used to determined beta diversity and visualized using principal coordinates analysis (PCoA). R version 3.5.1 (R Core Team, 2018) was used for all analyses. To tentatively assign taxonomy to the species level for differentially abundant OTUs, NCBI blastn using the nucleotide collection (nr/nt) database search was done. DESeq2 through the phyloseq package was used to determine differential abundances (DA) between groups (McMurdie and Holmes, 2013). When testing for differentially abundant OTUs by pet exposure, we also stratified the subjects into two age categories: being or under 58 year-old (≤58), or over 58 year-old (>58). Additionally, we performed a LEfSe (lindear discriminant analysis effect size) analysis on the Huttenhower Lab Galazy server (Segata et al., 2011). The phyloseq (McMurdie and Holmes, 2013), vegan (Oksanen et al., 2016), and ampvis2 (Anderson et al., 2018) packages as well as their dependencies were used for data visualization and statistical testing. All files can be found on figshare under https://doi.org/10.6084/m9.figshare.7764914.

### Statistical Analysis

ANOVA was used to test for differences in alpha diversity and PERMANOVA (Anderson, 2017) was used to test for significant differences in beta diversity. The Benjimini-Hochberg correction for the false discover rate was applied to all tests. The chi-square test and *t*-test were used to determine differences between those with and without pets. All statistical tests were considered significant with a *p* <0.05.

## Results

### Study Population

A total of 332 participants were included in the final analysis with 178 (53.6%) having some type of pet in the home. The most common type of pets in the home were dogs (*n* = 119, 67.2%) followed by cats (*n* = 79, 44.6%) and other pets including rodents, birds, and reptiles (*n* = 16, 9%). On average, there were 1.3 pets per household with a median of one (range:1–4 pets). Table 1 shows participant characteristics by pet exposure in the home. Pet owners were significantly younger than those without pets (*p* < 0.001). Race was also significantly different between the two groups (*p*-value: 0.031); however, there were no other significant differences between pet owners and those without pets.

**Table 1:** Population characteristics by exposure to household pets.

	**Overall** ***N* = 332**	**Pet in home** ***N* = 178**	**No pets in home** ***N* = 154**	***P-value***
Median Age	58 (18–94)	53 (18–93)	63 (19–94)	<0.001
Median BMI	29.25 (17.05–81.92)	29.55 (17.05–81.92)	28.92 (17.70–66.66)	0.13
**Race**				
Caucasian	285 (85.8%)	158 (88.8%)	127 (82.5%)	
African American	27 (8.1%)	8 (4.5%)	19 (12.3%)	
Other	20 (6.0%)	12 (6.7%)	8 (5.2%)	0.031
**Gender**				
Male	133 (40.1%)	70 (39.3%%)	63 (40.9%%)	
Female	199 (59.9%)	108 (60.7%%)	91 (59.1%%)	0.77
Median household size	2 (1–9)	2 (1–6)	2 (1–9)	
**Antibiotic taken in last year?**				
Yes	116 (36.8%%)	63 (37.7%%)	53 (35.8%%)	
No	199 (63.2%%)	104 (62.3%%)	95 (64.2%%)	0.73
**Recent Isolation in hospital**				
Yes	11 (3.3%)	7 (3.9%)	4 (2.6%)	
No	308 (92.8%)	164 (92.1%)	144 (93.5%)	
No response	13 (3.9%)	7 (3.9%)	6 (3.9%)	0.79
**Lower GI condition**				
Yes	101 (30.4%%)	54 (30.3%%)	47 (30.5%%)	
No	231 (69.6%%)	124 (69.7%%)	107 (69.5%%)	0.97
**Livestock Exposure**				
Yes	27 (8.2%%)	14 (7.9%%)	13 (8.6%%)	
No	303 (91.8%%)	164 (92.1%%)	139 (91.4%%)	0.82
**MDRO present in the gut**				
Yes	54 (16.3%%)	23 (13.0%%)	31 (20.1%%)	
No	277 (83.7%%)	154 (87.0%%)	123 (79.9%%)	0.079
**Take Probiotics in last year?**				
Yes	28 (8.9%%)	15 (8.7%%)	13 (9.0%%)	
No	288 (91.1%%)	157 (91.3%%)	131 (91.0%%)	0.92

### Composition and Diversity of the Gut Microbiota

**Figure 1:**
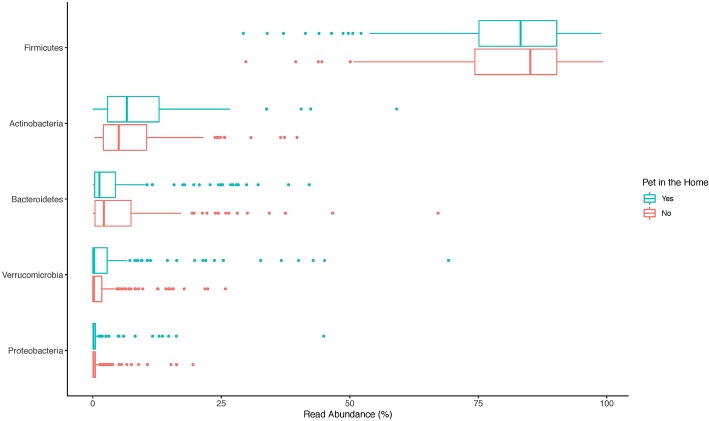
Boxplot of the relative read abundances of the top 5 phyla present in the gut microbiota.

**Figure 2:**
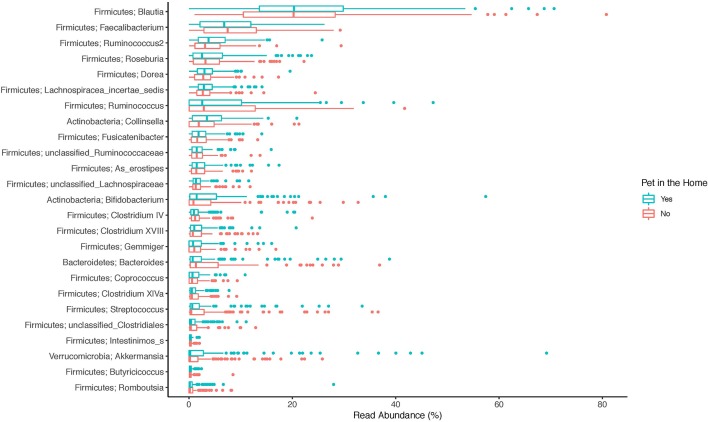
Boxplot of the relative read abundances of the top 25 genera present in the gut microbiota.

**Figure 3:**
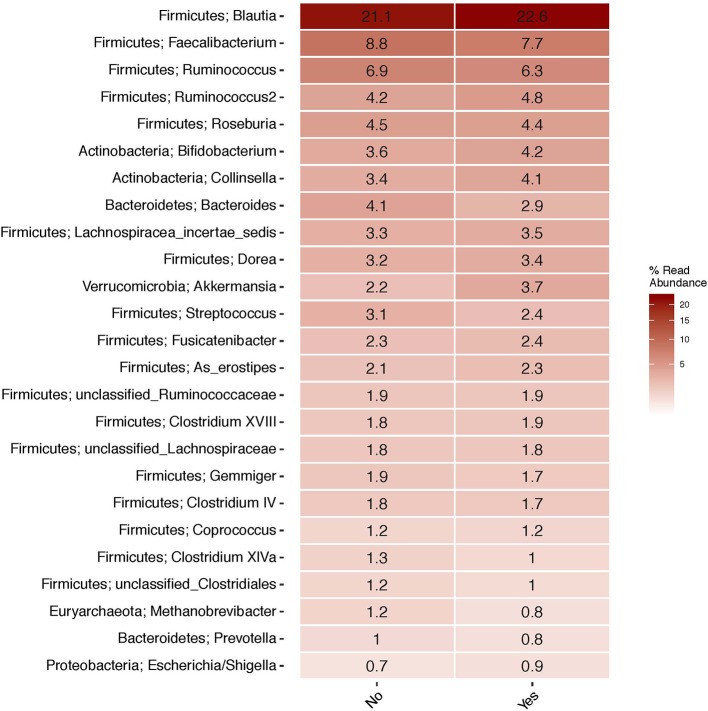
Heatmap of the top 25 most abundant genera in the population by pet exposure (yes/no).

The top 20 genera of bacteria identified in the stools of participants are shown in Figures 1–3. The gut microbiome of both pet owners and those without pets was dominated by the Firmicutes, which consisted of over 75% of the sequence reads. Of the Firmicutes, *Blautia* was the most prevalent genus with a little over 20% of the sequence reads in each group. Actinobacteria and Bacteroidetes were the next most prevalent phyla.

**Figure 4:**
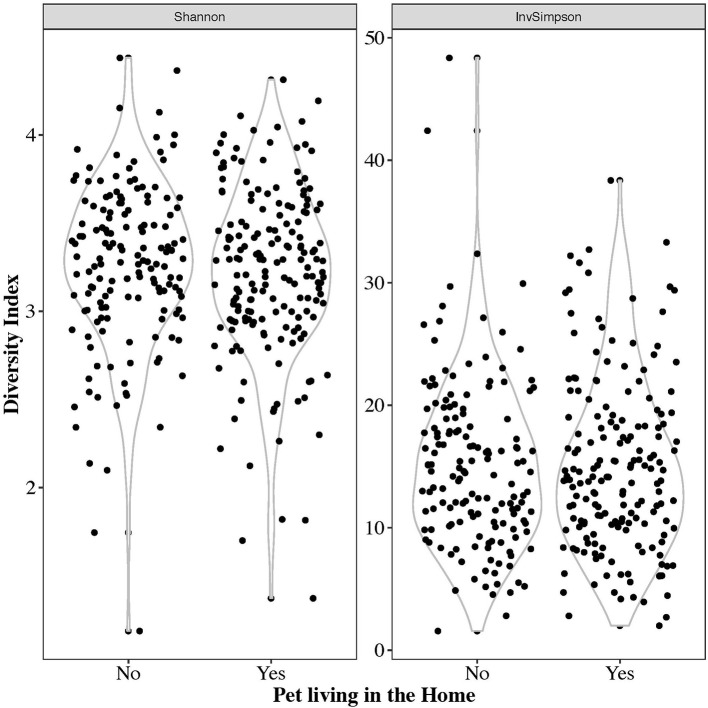
Violin plots of the Shannon and inverse Simpson's alpha diversity metrics on all 332 samples.

The alpha diversity of the gut microbiome, as represented by the Shannon and Inverse Simpson's metrics, is shown in Figure 4. The mean Shannon alpha diversity score was 3.28 with a range of 1.19–4.43. For the Inverse Simpson's method, the median score was 14.47 with a range of 1.58–48.36. No significant difference in alpha diversity indices was observed between those with pets and those without (Shannon *p*-value: 0.294, Inverse Simpson *p*-value: 0.23).

**Figure 5:**
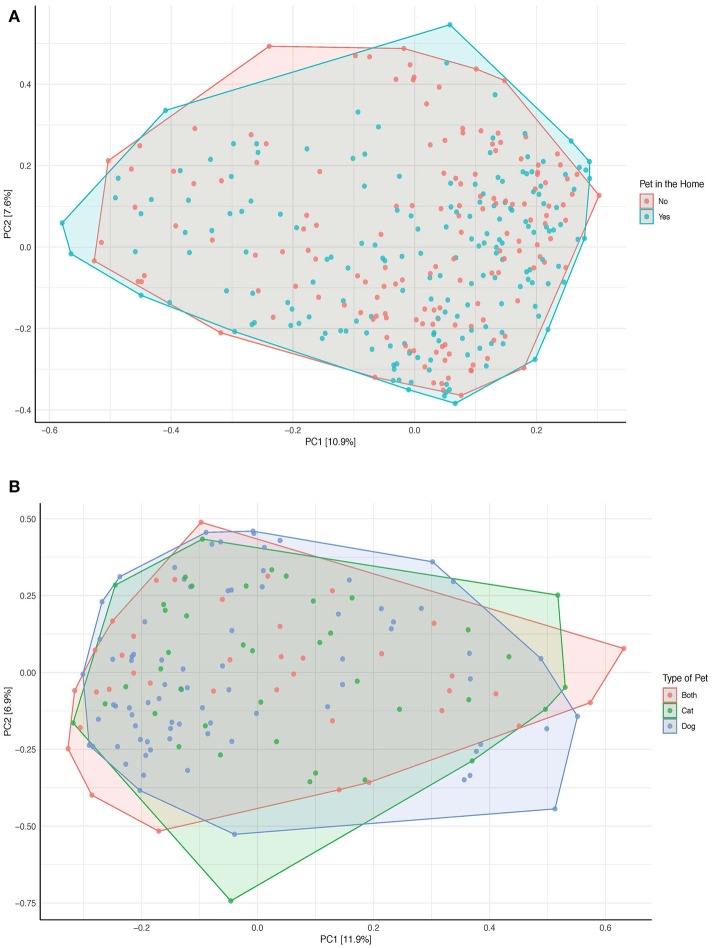
Principal Coordinates Analysis of the bray-curtis dissimilarity index **(A)** by pet exposure (*n* = 332). **(B)** By type of pet (either cat, dog, or both; *n* = 160). OTUs no present in more than 0.1% relative abundance in any sample have been removed. The data has been initially transformed by applying the hellinger transformation. The relative contribution (eigenvalue) of each axis to the total inertia in the data is indicated in the percent values included in each of the axis titles.

As seen in the Figure 5A, there are no discernable clusters in beta diversity by pet exposure and there is no difference in the overall species composition and beta diversity of those with pets and those without pets (Adonis *p*-value:0.137, test of homogeneity *p*-value: 0.317). Similar to the PCoA of all samples, there are no discernable clusters by the type of pet (Adonis *p*-value: 0.748, test of homogeneity *p*-value: 0.772; Figure 5B).

### Differentially Abundant OTUs Between Those With and Without Pets

**Figure 6:**
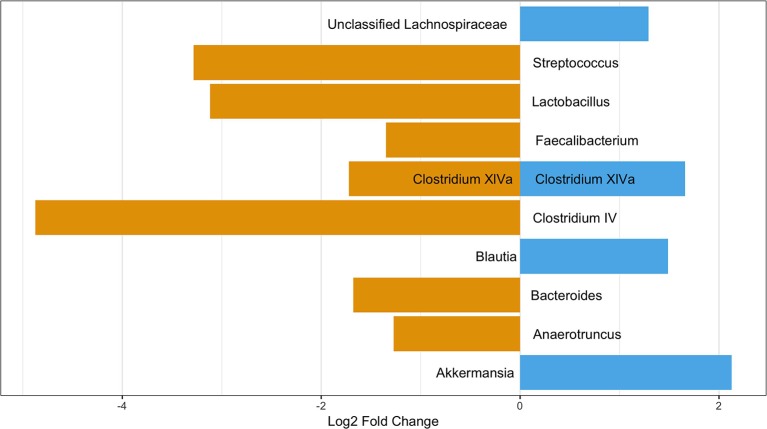
OTUs significantly differentially abundant between those with and without pets. Positive values represent OTUs more abundant in those with pets and negative values represent OTUs more abundant in those with not pets. The Benjimini-Hochberg correction for the false discover rate was applied.

Although there were no significant differences in alpha or beta diversity between those with and without pets, there were 11 OTUs differentially abundant between participants with pets in the home and those without (Figure 6). Four OTUs were more abundant in those participants with a pet and seven OTUs were more abundant in those without pets in the home. All but two OTUs belonged to the phylum Firmicutes with one belonging to the phylum Verrucomicrobia and the other to the Bacteroidetes. Four OTUs were tentatively classified to the species level using the NCBI blastn function: *Lactobacillus gasseri* (OTU 65)*, Clostridium oroticum* (OTU 1089)*, Bacteroides cellulosilyticus* (OTU 120), and *Akkermansia muciniphila* (OTU 784)*. L. gasseri* and *B. cellulosilyticus* were more prevalent in those with no pets while *C. oroticum* and *A. muciniphila* were more prevalent in those with pets in the home. Of these OTUs, seven were potentially present due to confounding variables (antibiotic use in the last year, probiotic consumption, and contact with healthcare). The analyses of these three confounders can be found in the Supplemental Material. After considering these confounders, the following OTUs remained significantly different between those with and without pets: OTU 219 (*Clostridium XlVa*), 303 (*Anaerotruncus*), 120 (*Bacteroides*), and 784 (*Akkermansia*). When considering only cats and dogs, OTU 784 was not significant (data not shown). The LEfSe analysis identified 8 OTUs associated with no pet exposure and 3 with pet exposure (Figure S8). Of these, the only common OTU was OTU 120 (*Bacteroides*) which was significantly more abundant in those without pets in all analyses; however, this OTU was identified in the negative control samples as a contaminant (S1. Text).

**Figure 7:**
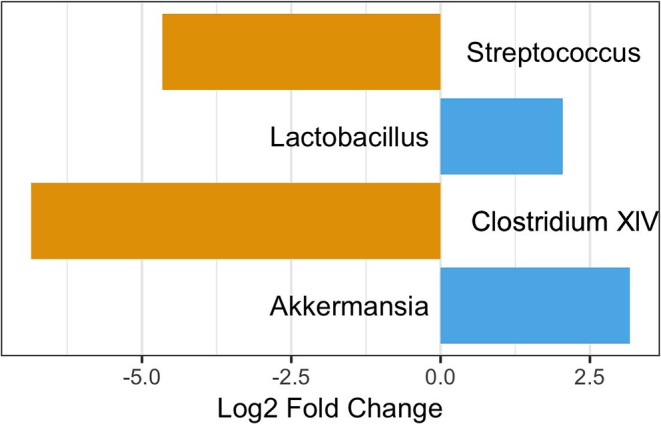
OTUs significantly differentially abundant between those with and without pets age 58 and under. Positive values represent OTUs more abundant in those with pets and negative values represent OTUs more abundant in those with not pets. The Benjimini-Hochberg correction for the false discover rate was applied.

**Figure 8:**
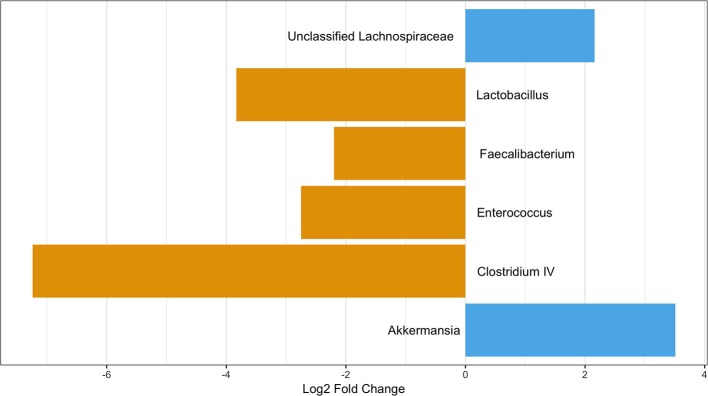
OTUs significantly differentially abundant between those with and without pets over age 58. Positive values represent OTUs more abundant in those with pets and negative values represent OTUs more abundant in those with not pets. The Benjimini-Hochberg correction for the false discover rate was applied.

As age was significantly different between those with and without pets (Table 1), we performed a differential abundance test while also stratifying by age category to ensure any differences were not due to age. When considering those participance age 58 and under, 3 OTUs were more abundant in those with pets compared to those without: *Akkermansia, Lactobacillus, and Clostridium XIVa*. None of these were OTUs seen in the differential abundance analysis on all participants. We also observed two OTUs, a *Clostridium XIVa* and a *Streptococcus*, that were more abundant in those without pets. These were the same OTUs as observed in the differential abundance analysis on all participants (Figure 7). In the over 58 age group stratification, we observed two OTUs—*Akkermansia* and an unclassified *Lachnospiraceae*—that were more abundant in those with pets and four OTUs—*Faecalibacterium, Enterococcus, Lactobacillus*, and *Clostridium IV*—that were more abundant in participants with no pets. Of these In the over 58 age group stratification, only the *Enterococcus* OTU was newly identified (Figure 8).

## Discussion

Here we have characterized the impact of pet exposure on the gut microbiota of a cohort of adults in Wisconsin using 16S rRNA sequencing. Within our cohort, there was little difference in the diversity and composition of the gut microbiota by pet exposure, though there were several OTUs that were differentially abundant based on pet exposure. The results indicate there is likely little impact of owning a pet on the composition of the adult gut microbiota.

While there is limited research on the impact of pet ownership on the microbiome, prior research has demonstrated an impact of pet exposure on other adult microbiomes, such as the skin, with the dog ownership significantly altering the microbiome (Song et al., 2013). Others have found pet ownership increases diversity across multiple environmental microbiomes within the home and have higher relative abundances of dog-associated taxa which may impact the human microbiome as well (Dunn et al., 2013). Several culture-based studies have also demonstrated shared gut bacteria between humans and their pets (Damborg et al., 2009).

Though prior research has demonstrated an impact of pets on microbial communities, one reason we may not have observed an impact on diversity is that pet exposure may have a greater effect on the gut microbiome earlier in life, where pet exposure has been shown to impact the gut microbiome in several studies (Azad et al., 2013; Nermes et al., 2015). Studies examining the effect of age on the gut microbiome have found decreases in interpersonal variation with increasing age (Palmer et al., 2007; Yatsunenko et al., 2012) and have found that the gut microbiome begins to reflect the adult gut microbiome by 3 or 4 years of age (Yatsunenko et al., 2012; Bergstrom et al., 2014); though other studies have shown the gut microbiome undergoes constant changes in composition over a lifetime (Odamaki et al., 2016). It may be possible the gut microbiome is most susceptible to alterations by pets at an early age, though no conclusions can be reached without additional research on the pet exposure across differing age groups.

In our study, the gut microbiota was heavily dominated by members of the phylum Firmicutes, followed by a much lower relative abundance of Actinobacteria and Bacteroidetes. This is in contrast to other studies, like the Human Microbiome Project (HMP), which have shown that Bacteroidetes and Firmicutes are the most prevalent phyla in the gut (Consortium, 2012). There are several potential explanations for this difference. The first is due to DNA extraction method. The method used in this study was designed to improve lysis of Gram-positive bacteria by adding lysozyme, lysostaphin, and mutanolysin to a bead-beating protocol. Many studies on the gut microbiome have lacked appropriate enzymes to lyse Gram-positive cell walls, resulting in an underrepresentation of these bacteria (Bork et al., 2015), including the Firmicutes phylum. Other studies have found fecal collection methods may create bias by the Gram-status of the organism with Gram-positive organisms being more prevalent in fresh, frozen stools compared to commercially available collection tube kits (Watson et al., 2019). Samples for this study were not frozen until arrival in the lab (<72 h after collection), but were collected fresh and shipped at 4°C with no buffers. One additional, though less likely, explanation is the majority of participants in the study fell into the same community type. The HMP project identified four common stool community types with community type B being dominated by members of the Firmicutes phylum and low amounts of Bacteroidetes, similar to what we observed in this study, although this community type made up <10% of the HMP samples (Ding and Schloss, 2014).

Our study identified 4 OTUs more abundant in those with pets and 7 OTUs more abundant in those without pets. However, when we stratified this analysis by age, four of these OTUs—*Clostridium* (OTU1089), *Blautia* (OTU 411), *Anaerotruncus* (OTU 303), *and Bacteroides* (OTU 120)—were no longer significantly different, implying that these OTUs may have differed due to age and not pet exposure. Furthermore, several OTUs identified in our stratification analysis were not seen when considering the entire cohort together. It should also be noted two OTUs identified as differentially abundant were observed in the negative control samples and any difference in these OTUs (OTU 105, OTU120) is likely due to contamination. No other OTUs identified as differentially abundant were observed in the negative control samples (see S1. Text). Additional analyses of antibiotic use, probiotic consumption, and contact with healthcare showed that several of the OTUs identified as differentially abundant may have been due to the confounders. After accounting for these variables, four OTUs remaining differentially abundant (OTUS 120, 219, 303, and 784), though only OTU 219 and 784 remain after accounting for age. In humans, *Clostridium XIVa* is associated with short chain fatty acid production and are thought to help prevent infections in the gut. *Clostridium* XIVa (OTU 219) has also been found in the guts of cats and dogs (Suchodolski, 2011). We hypothesize humans may potentially come into contact with thus gut associated OTU in several ways. The first would be during cleaning up the pet's fecal material, though it may also be possible the animals transfer this organism from their tongues after the animal grooms itself.

OTU 784, *Akkermansia*, was not present when only assessing the microbiome of cat and dog owners, though this genera has previously been identified in rodents (Nagpal et al., 2018) and reptiles (Campos et al., 2018). *Akkermansia* was the only differentially abundant OTU we were able to tentatively to the species level. OTU 784 identified as *Akkermansia muciniphila*, a Gram-negative anaerobe commonly found in the human gut and thought to protect against pathogen invasion via competitive inhibition (Belzer and de Vos, 2012; Derrien et al., 2017). Moreover, it is thought to restore intestinal mucin (Derrien et al., 2017) and its presence in low levels may indicate a thin mucous layer (Brahe et al., 2015). *A. muciniphila* typically decreases with age and is lower in those suffering with Inflammatory Bowel Disease and obesity. Interestingly, this OTU was identified in those over 58 with pets. Additionally, those with pets were no more likely to have lower gastrointestinal conditions and had a median BMI that was considered overweight (Anhê et al., 2015; Brahe et al., 2015).

Although we were not able to assess the pet microbiome in this study, others have characterized the gut microbiome of both cats and dogs. The gut of cats is typically dominated by Firmicutes and Bacteroidetes (Deng and Swanson, 2015), while the canine gut microbiome is characterized by Firmicutes, Proteobacteria, Fusobacteria, and Bacteroidetes (Deng and Swanson, 2015). In our study, Firmicutes was commonly found in all participants, although Bacteroidetes was found in very low levels and Proteobacteria and Fusobacteria were <1% of all phyla.

Our current study has several strengths including being a part of a large, statewide microbiota study from a general population. Additionally, we used an extraction method that is able to capture difficult to lyse bacteria to more accurately represent the gut microbiome. However, our study also has limitations. The design of the SHOW and Wisconsin Microbiome projects is cross-sectional, so our analysis was limited to a single snapshot in time. As such, we cannot assess how changes in pet exposure or other factors may relate to the composition of the gut microbiota longitudinally. Second, pets were not sampled as part of the SHOW or the Wisconsin Microbiome Project and thus we were unable to compare each participant's microbiota with their pet's microbiota. Additionally, no children were included in this analysis and as such, we were unable to determine if the impact of pet exposure is different by participant age. Lastly, we were only able to assess the 16S rRNA gene, which does not allow us to determine if there are functional differences in the microbiome as a result of pet exposure. Use of the 16S rRNA is also limited in its ability to reliably assign taxonomy down to the species level, as can be more easily accomplished using other methods like shotgun metagenomics.

## Conclusions and Future Directions

In this study, we found very few differences in the gut microbiota of participants based on exposure to household pets. While there was no difference in alpha or beta diversity, there were two OTUs differentially abundant in those with pets compared to those without after assessing potential confounding variables. Future research is needed to further elucidate the relationship between the gut microbiome and pets. Future studies should take a longitudinal perspective and assess the impact of age on this relationship. Pets may serve as a reservoir for potential pathogens as well as reduce the likelihood of atopic diseases. As such, understanding the relationship between our microbiomes and our pets is fundamental for elucidating the role bacteria play in human health.

## Data Availability Statement

The datasets analyzed for this study can be found on figshare under https://doi.org/10.6084/m9.figshare.7764914.

## Ethics Statement

The studies involving human participants were reviewed and approved by University of Wisconsin-Madison Institutional Review Board. The patients/participants provided their written informed consent to participate in this study.

## Author Contributions

AK, OJ, AS, MD, KP, GS, and NS contributed to the conception and design of the study. LW and JS extracted DNA and carried out the sequencing for this study. AK, OJ, MD, and LW created, cleaned, and organized the databases used for the study. AK and OJ carried out the microbiota and statistical analyses with oversight from AS, GS, and NS. AK wrote the first draft of the manuscript. All authors contributed to manuscript revision, read, and approved the final submitted version of the manuscript.

### Conflict of Interest

The authors declare that the research was conducted in the absence of any commercial or financial relationships that could be construed as a potential conflict of interest.
