# Supplementary material for: Household Pet Ownership and the Microbial Diversity of the Human Gut Microbiota
Source: Front Cell Infect Microbiol. 2020 Feb 28;10:73. doi: 10.3389/fcimb.2020.00073 (PMC7058978; doi:10.3389/fcimb.2020.00073)
Supplement: Supplementary file 1 [file Data_Sheet_1.docx]

**S1. Text. Description and characterization of the negative control samples.**

**S1 Additional Methods:**

Negative controls were used for every set of DNA extractions, each PCR run, and during sequencing. DNA extraction negative controls consisted of only extraction reagents with PCR and sequencing negative controls consisting of the PCR and sequencing reagents plus DNAse, RNAse free water. A total of 22 negative controls were used

**S2 Analysis of the Negative Control Samples**

A majority of negative control samples had less than 10 reads (Fig S1). The negative controls clustered separately from the stool samples as seen in Fig S2. Several OTUs were identified in higher abundance in the negative control samples than in the stool samples (Fig S3). Lastly, two OTUs identified as differentially abundant were observed in the negative control samples and any difference in these OTUs (OTU 105, OTU120) may be due to contamination (Fig S4). No other OTUs identified as differentially abundant were observed in the negative control samples


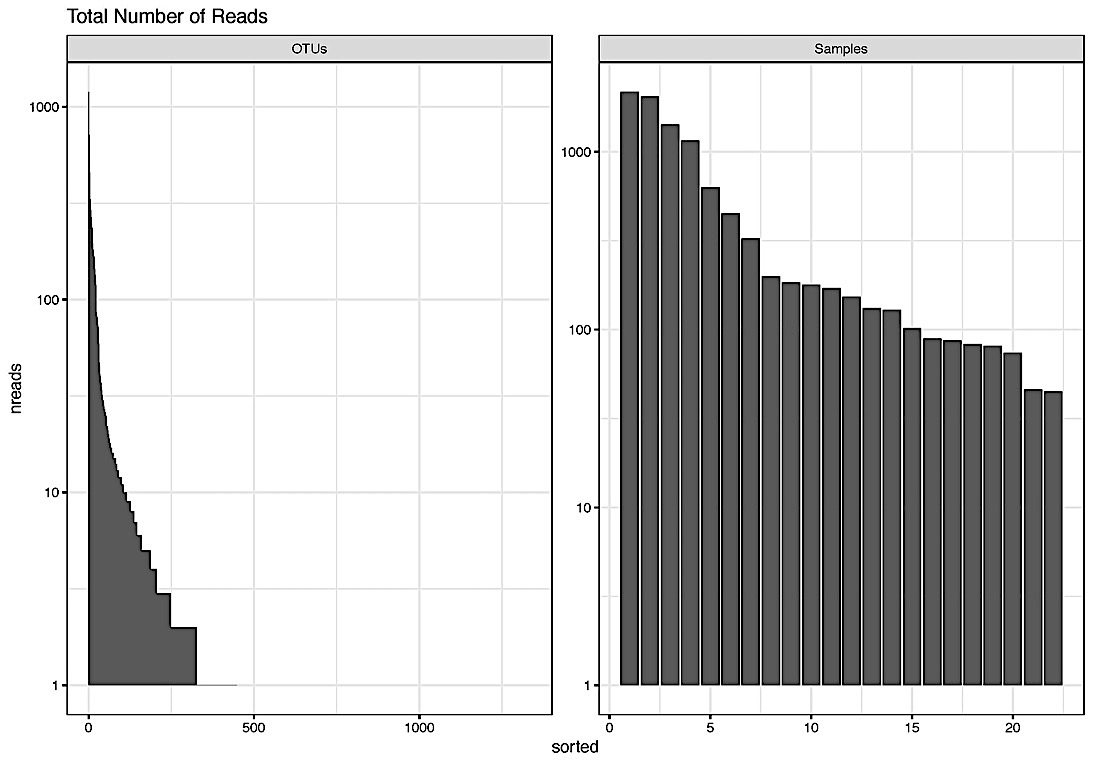


Figure S1. Histogram of the number of reads in the negative controls.


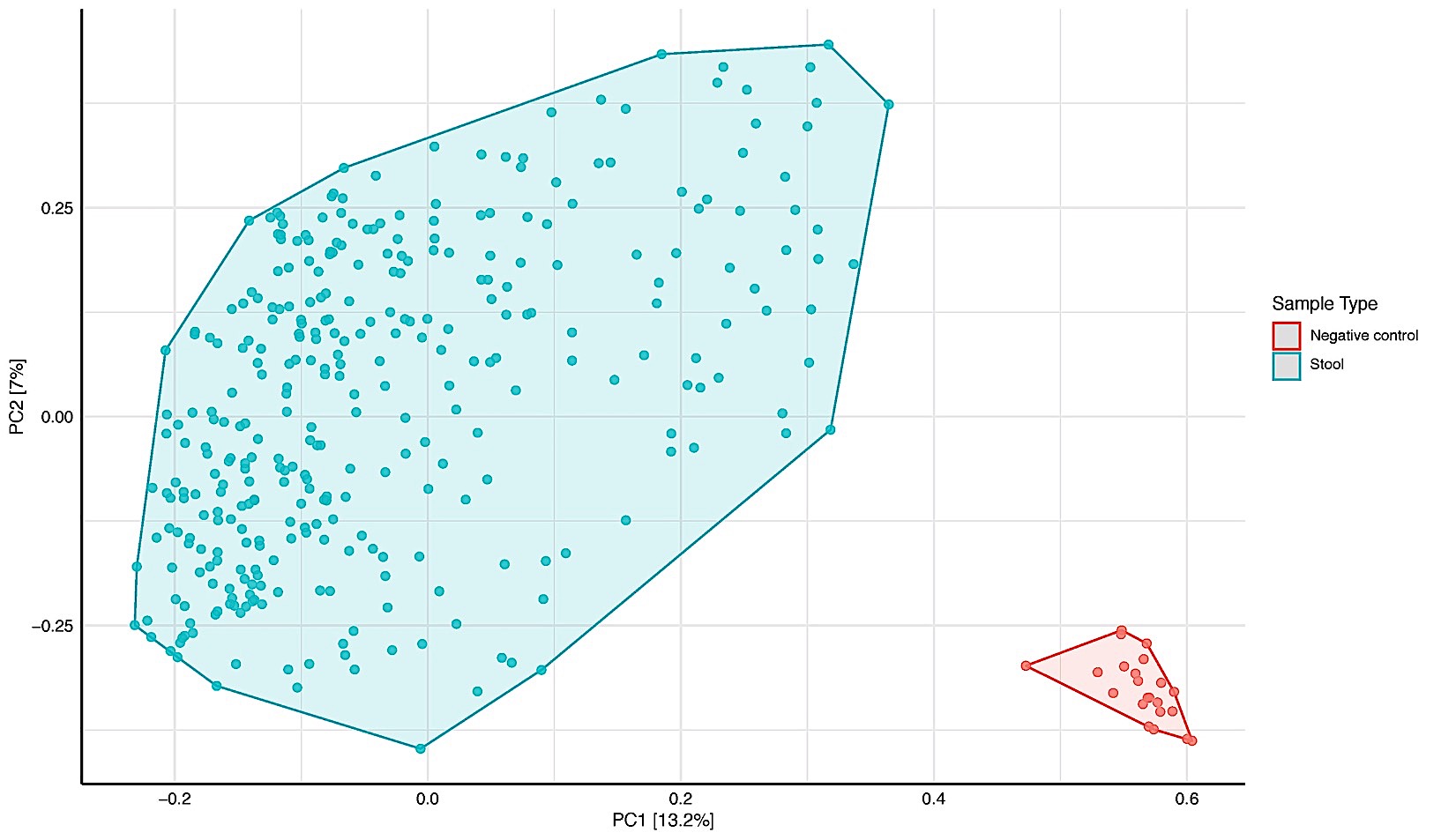


Figure S2. Principal Coordinates Analysis of the Bray-Curtis dissimilarity index by sample type. OTUs no present in more than 0.1% relative abundance in any sample have been removed. The data has been initially transformed by applying the Hellinger transformation. The relative contribution (eigenvalue) of each axis to the total inertia in the data is indicated in the percent values included in each of the axis titles.


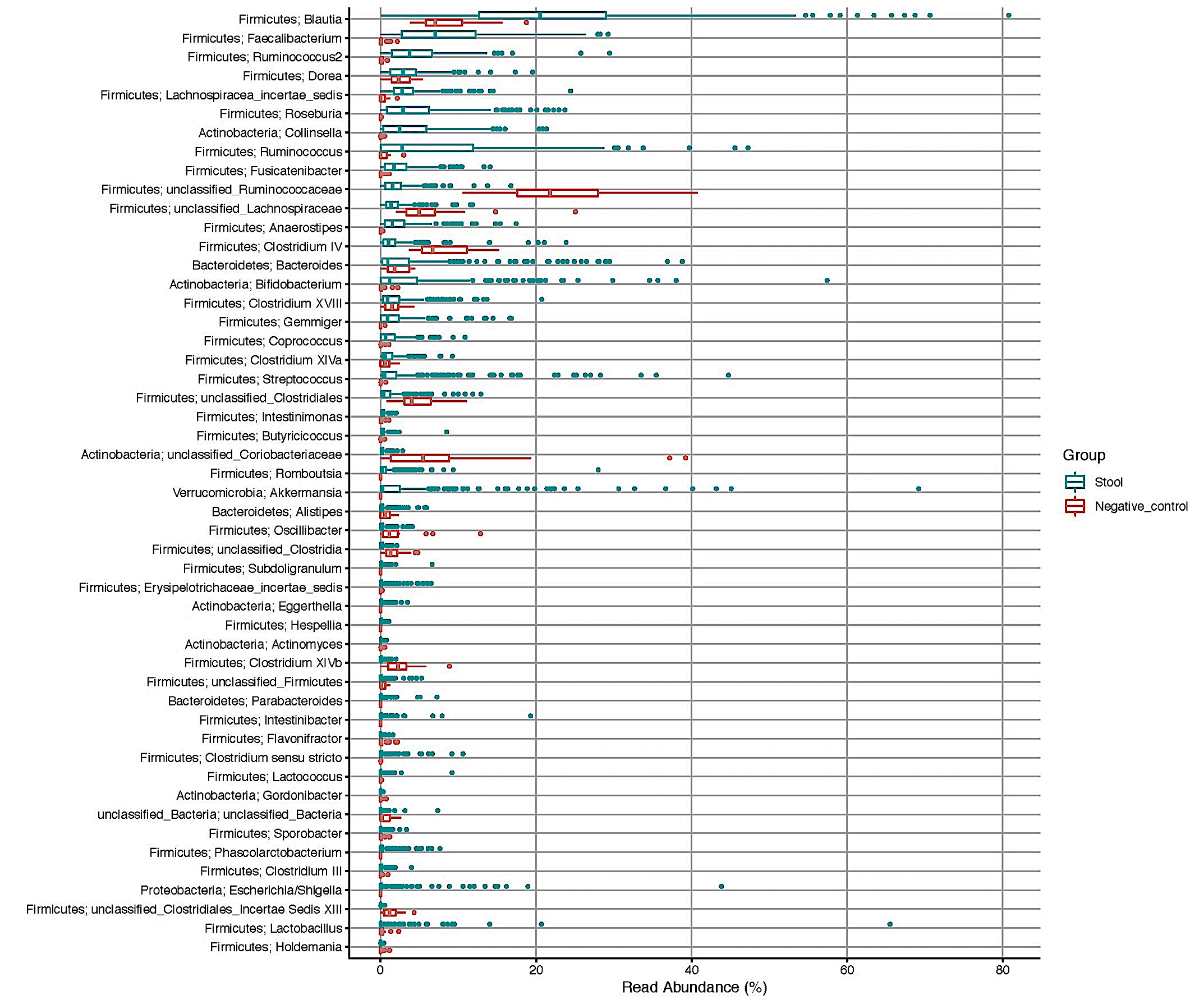


Figure S3. Boxplot of the top 50 OTUs observed in the negative control and stool samples.


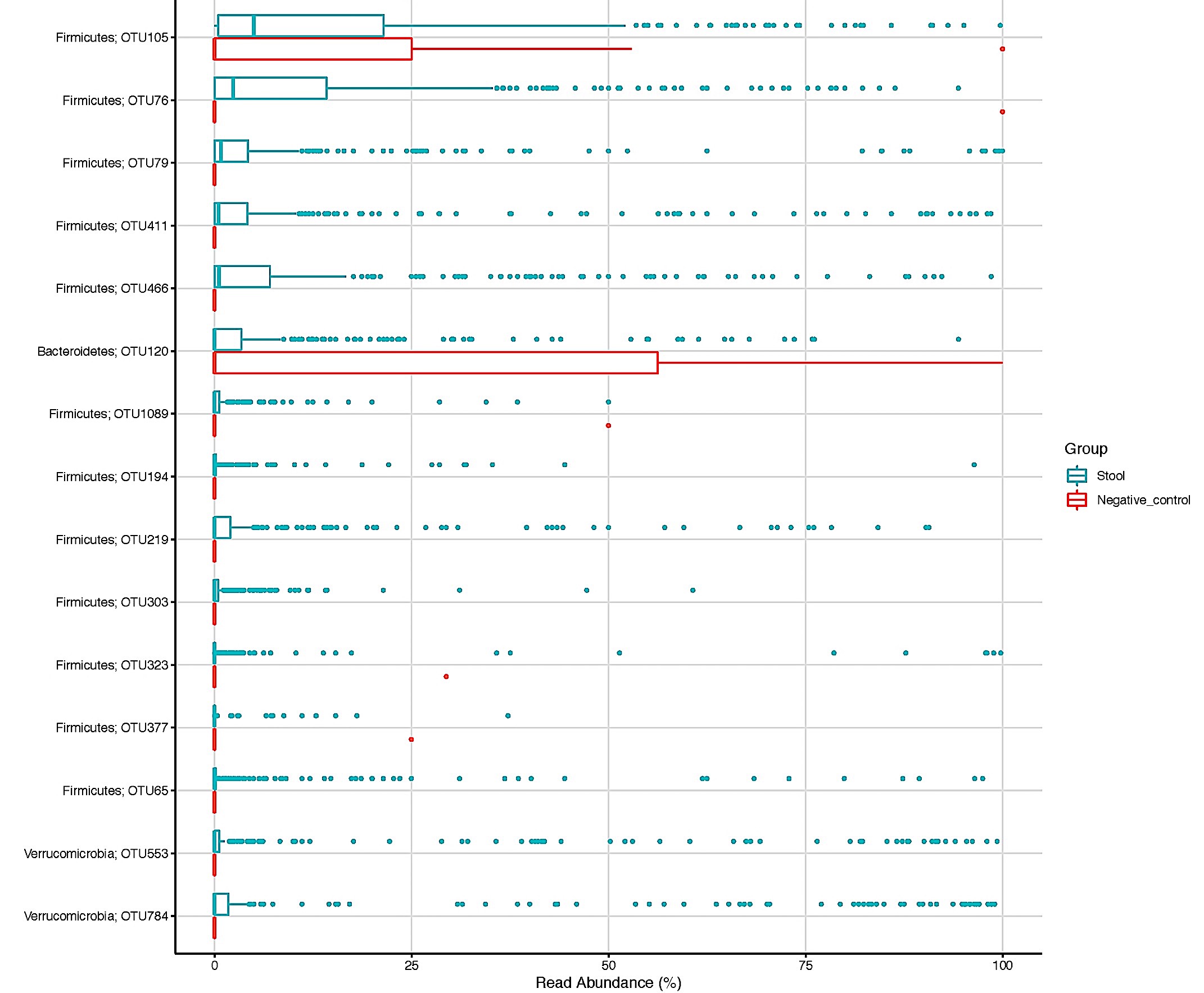
Figure S4. Boxplot of the OTUs identified in the differential abundance analysis comparing the stool samples and negative control samples.

**S3 Analysis of Antibiotic exposure, probiotic consumption, and healthcare exposure**

We compared those: who took antibiotics in the last year to those that did not; those who reported taking probiotics to those who did not; and those who reported some contact with healthcare (defined as visiting a hospital, ICU, or long-term care facility) to those who did not. There were no differences in alpha and beta diversity in any comparison. There were significantly differentially abundant OTUs identified by the DA analysis of antibiotics (Figure S5), probiotics (Figures S6), and healthcare exposure (Figure S7).


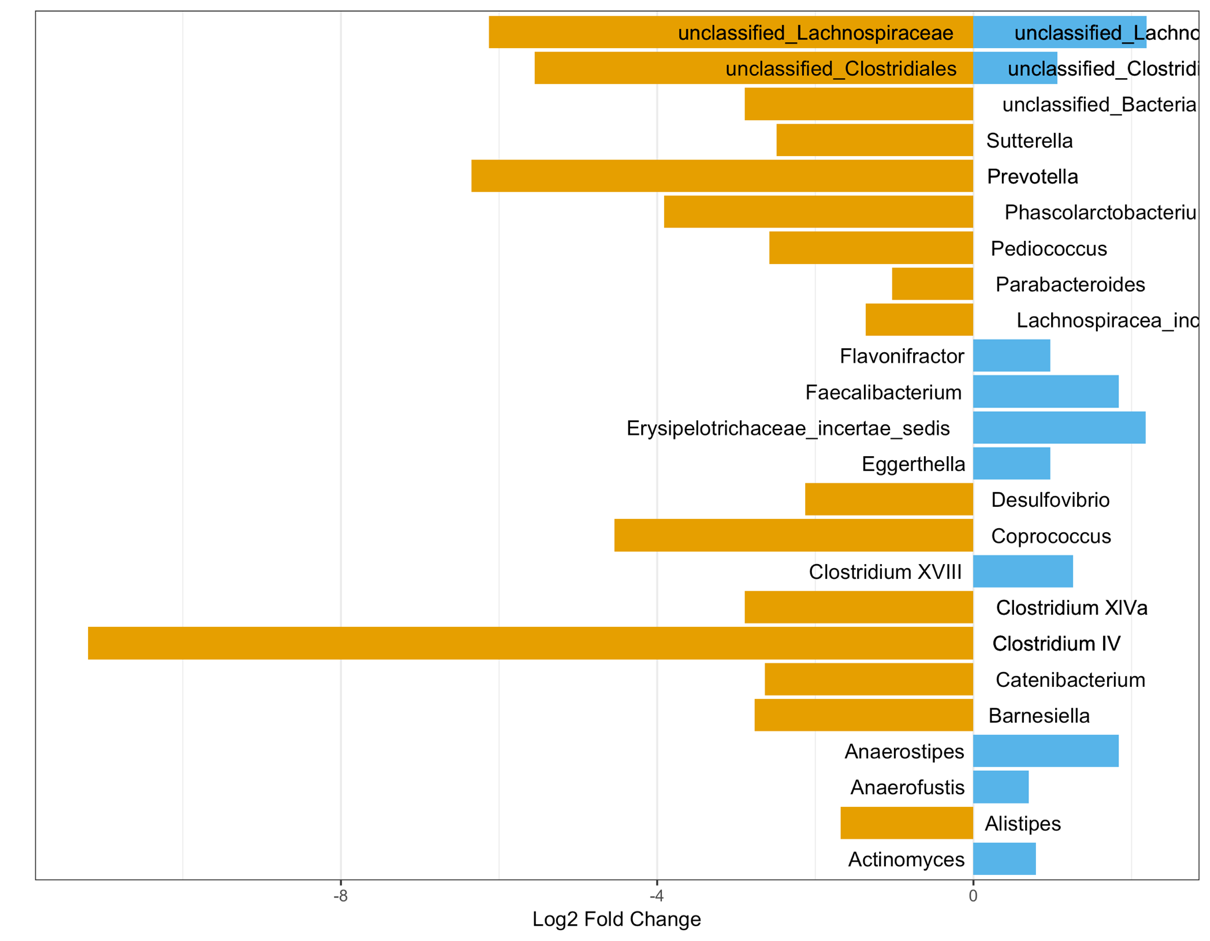


Figure S5. OTUs significantly differentially abundant between taking and not taking antibiotics. Positive values represent OTUs more abundant in those who took antibiotics and negative values represent OTUs more abundant in those who did not. The Benjimini-Hochberg correction for the false discover rate was applied.


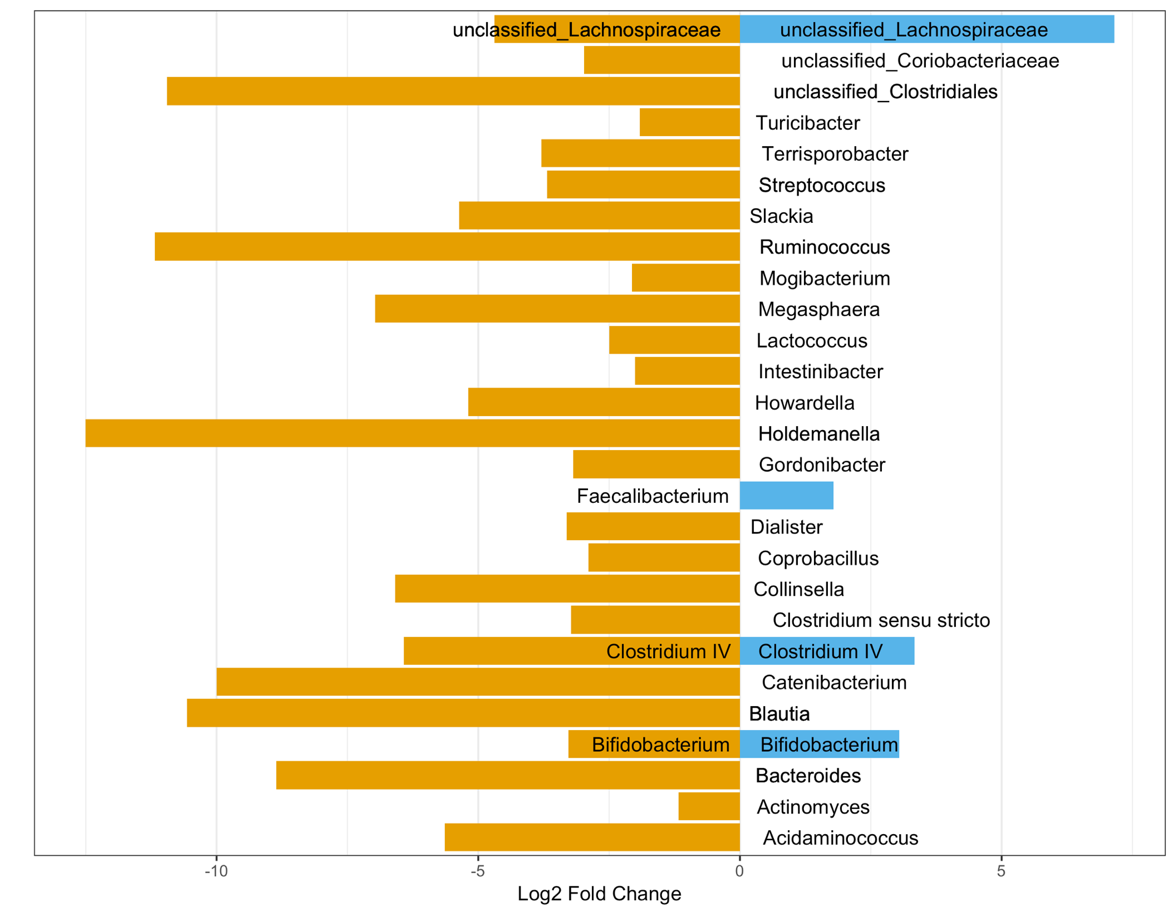


Figure S6. OTUs significantly differentially abundant between taking and not taking probiotics. Positive values represent OTUs more abundant in those who took probiotics and negative values represent OTUs more abundant in those who did not. The Benjimini-Hochberg correction for the false discover rate was applied.


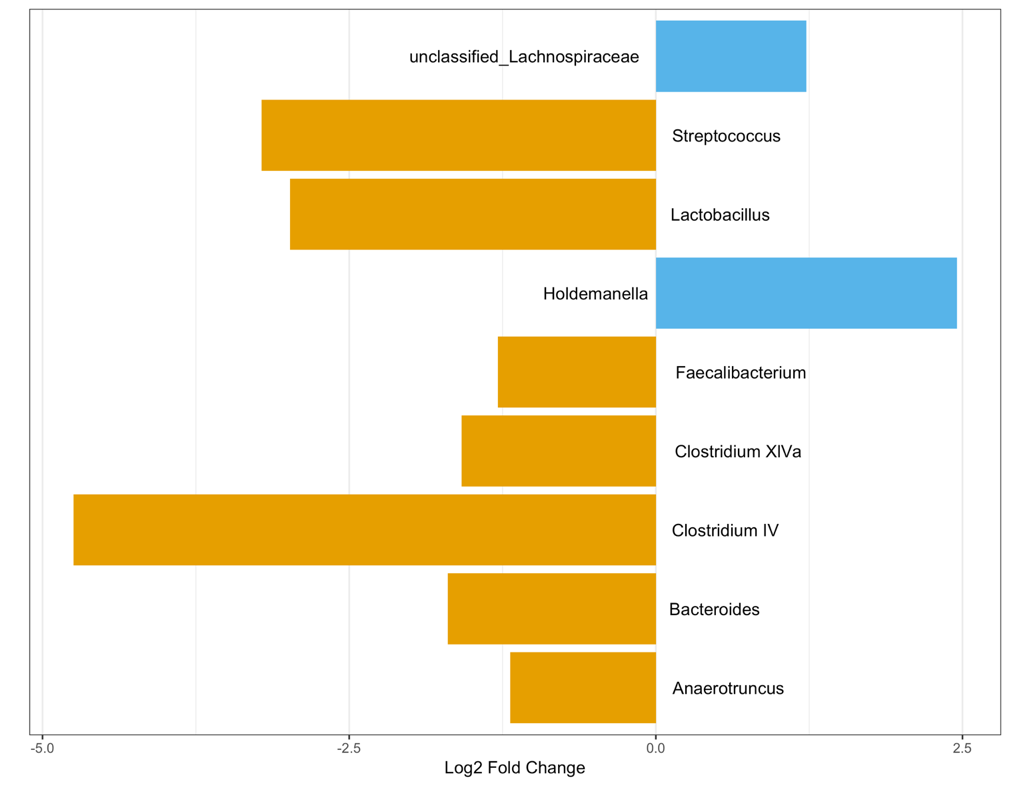


Figure S7. OTUs significantly differentially abundant between those with and without exposure to healthcare. Positive values represent OTUs more abundant in those who visited a healthcare facility and negative values represent OTUs more abundant in those who did not. The Benjimini-Hochberg correction for the false discover rate was applied.


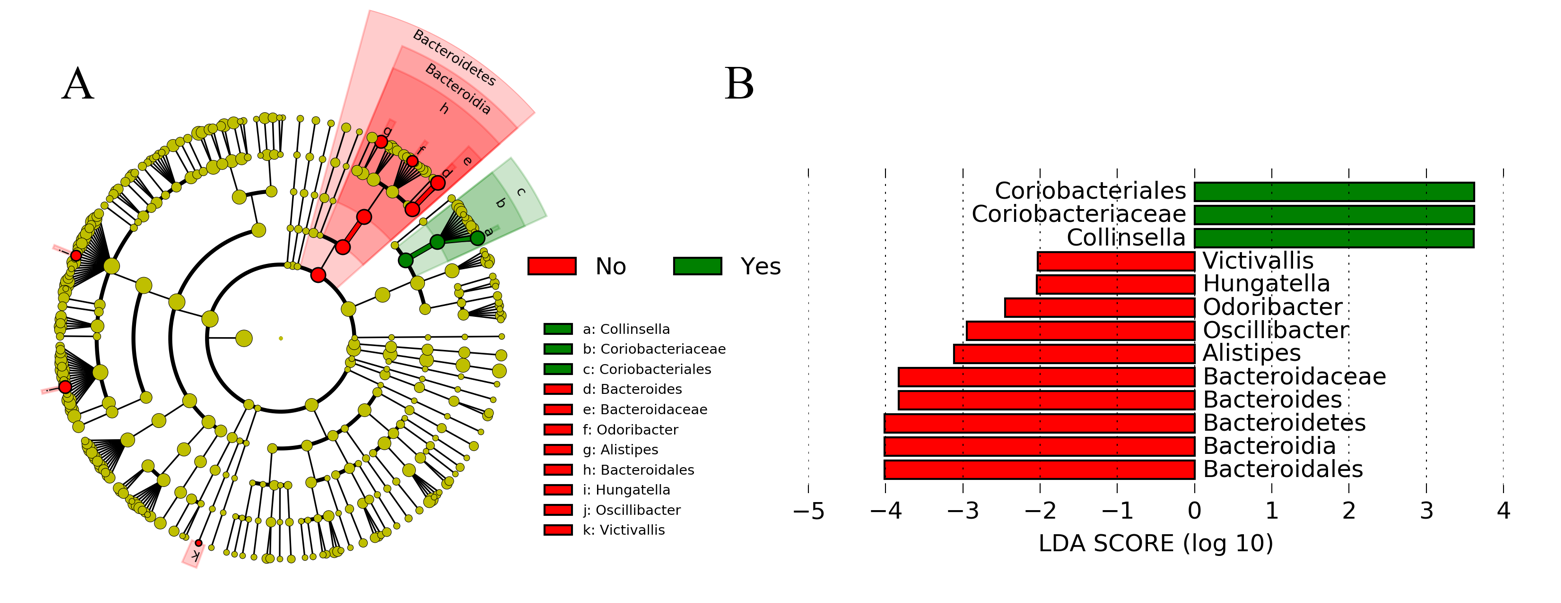
Figure S8. Cladogram (A) and barplot (B) representing the OTUs associated with pet exposure according to the LEfSe analysis.
